# Supplementary material for: Age-related changes in circadian regulation of the human plasma lipidome
Source: Commun Biol. 2023 Jul 20;6:756. doi: 10.1038/s42003-023-05102-8 (PMC10359364; doi:10.1038/s42003-023-05102-8)
Supplement: Supplementary file 2 — Supplemental Material [file 42003_2023_5102_MOESM2_ESM.pdf]

Age-related changes in circadian regulation of the human plasma lipidome

Shadab A. Rahman,[1,2,] Rose M. Gathungu,[1,2†] Vasant R. Marur,[1,2††] Melissa A. St. Hilaire,[1,2] Karine Scheuermaier, [1,2,†††] Marina Belenky,[1,2] Jackson S. Struble,[1,2] Charles A. Czeisler,[1,2] Steven W. Lockley,[1,2] Elizabeth B. Klerman,[1,2,3] Jeanne F. Duffy,[1,2] Bruce S. Kristal[1,2*]

[1]Division of Sleep and Circadian Disorders, Departments of Medicine and Neurology, Brigham and Women's Hospital, 221 Longwood Ave, Boston, MA 02115, USA;
[2]Division of Sleep Medicine, Harvard Medical School, Boston, MA 02115, USA;
[3]Department of Neurology, Massachusetts General Hospital, Boston MA 02114, USA.

*CORRESPONDING AUTHOR: Bruce S. Kristal - Email: bkristal@bwh.harvard.edu

†Present Address: Enara Bio, The Magdalen Centre, Oxford Science Park, 1 Robert Robinson Avenue, Oxford, OX4 4GA, UK

††Present Address: Quantitative Biosciences, Merck & Co., Inc, 320 Bent St, Cambridge, MA 02141

†††Present Address: Brain Function Research Group, School of Physiology, Faculty of Health Sciences, University of the Witwatersrand, 7 York Road, Parktown 2193, Johannesburg, South Africa

Supplementary information

Supplementary Tables

Supplementary Figures and Legends

Supplementary Table 1

ID	Age (years)	Sex	Wake Time (h:mm)	Bed Time (h:mm)	$DLMO_{25\%}$ (h:mm)
28E5H	59	MALE	6:01	22:01	19:31
28J7H	56	MALE	5:28	21:28	20:07
27D5H	69	MALE	5:01	21:01	20:05
2902H	55	FEMALE	7:01	23:01	22:01
2906H	56	MALE	5:18	21:18	20:46
2930H	56	MALE	6:01	22:01	19:00
2947H	64	FEMALE	7:57	23:57	23:04
2954H	58	MALE	6:40	22:40	20:48
28G1H	57	FEMALE	7:58	23:58	20:36
29V8H	57	FEMALE	6:07	22:07	19:19
29V4H	55	FEMALE	7:21	23:21	22:01
28Q5H	57	MALE	7:01	23:01	21:58
3003V	31	MALE	6:59	22:59	22:20
3037V	28	MALE	8:29	0:29	22:17
3057V	20	MALE	9:37	1:37	23:52
3060V	21	MALE	7:29	23:29	21:14
3074V	23	MALE	8:02	0:02	20:49
3079V	25	MALE	7:59	23:59	21:21
3081V	23	FEMALE	6:35	22:35	20:33
3085V	21	MALE	8:10	0:10	20:13
3091V	20	MALE	8:33	0:33	21:32
3113V	20	FEMALE	7:31	23:31	21:00
3121V	30	FEMALE	5:44	21:44	21:21
3129V	21	FEMALE	9:54	1:54	23:35

Supplementary Figure 1

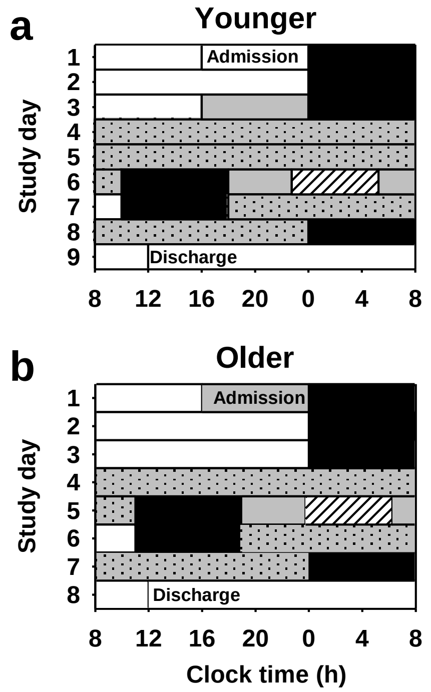

Supplementary Figure 1. Study protocol for assessing circadian rhythms in the human plasma lipidome. All study events were timed according to each individual's schedule, which was maintained for at least three weeks prior to starting the inpatient part of the study. Example study raster for an individual with habitual self-selected sleep between midnight and 8:00 h in the (a) younger and (b) older age groups. Black bars represent scheduled sleep in darkness (time in bed), and white bars represent being awake with indoor intensity light [~0.23 W/m^2 (~88 lux)] when measured in the vertical plane at a height of 137 cm]. Gray bars represent being awake in dim light condition [older: ~0.0087 W/m^2 (~3.3 lux)]; younger: ~0.001 W/m^2 (~0.5 lux), when measured in the vertical plane at a height of 137 cm], and gray dotted bars represent constant routine intervals in dim lighting. Hashed bars represent the light exposure intervention. Only data from the first 27 hours of the first constant routine for both groups was included in the current study.

Supplementary Figure 2

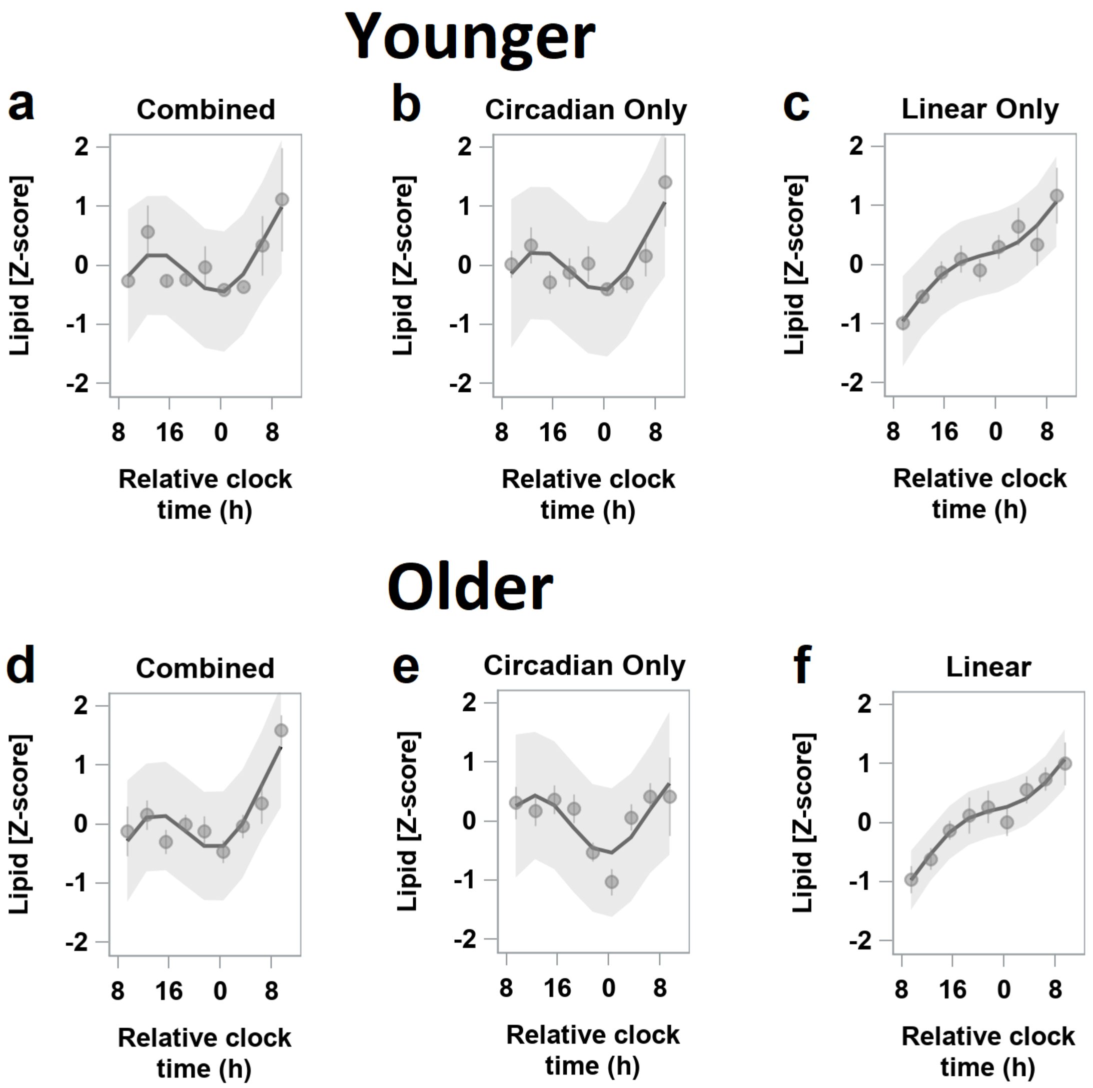

Supplementary Figure 2. Example time-course profile of a lipid species best modelled with a Cosinor regression model in which the significant components were both the 24-h (sinusoidal) harmonic and the linear component (combined), only the sinusoidal component (circadian only), or only the linear component (linear only) in the younger (a, b, c) and older (d, e, f) groups, respectively. Group-mean estimates (±SE) of the Z-score transformed data in each 3-h time bin during the 27-h constant routine are shown (●, gray filled circle). Solid black line represents the lipid concentration predicted by the Coinor regression. The 95% confidence interval (CI) region of the regression is shown as the gray shaded band.

Supplementary Figure 3

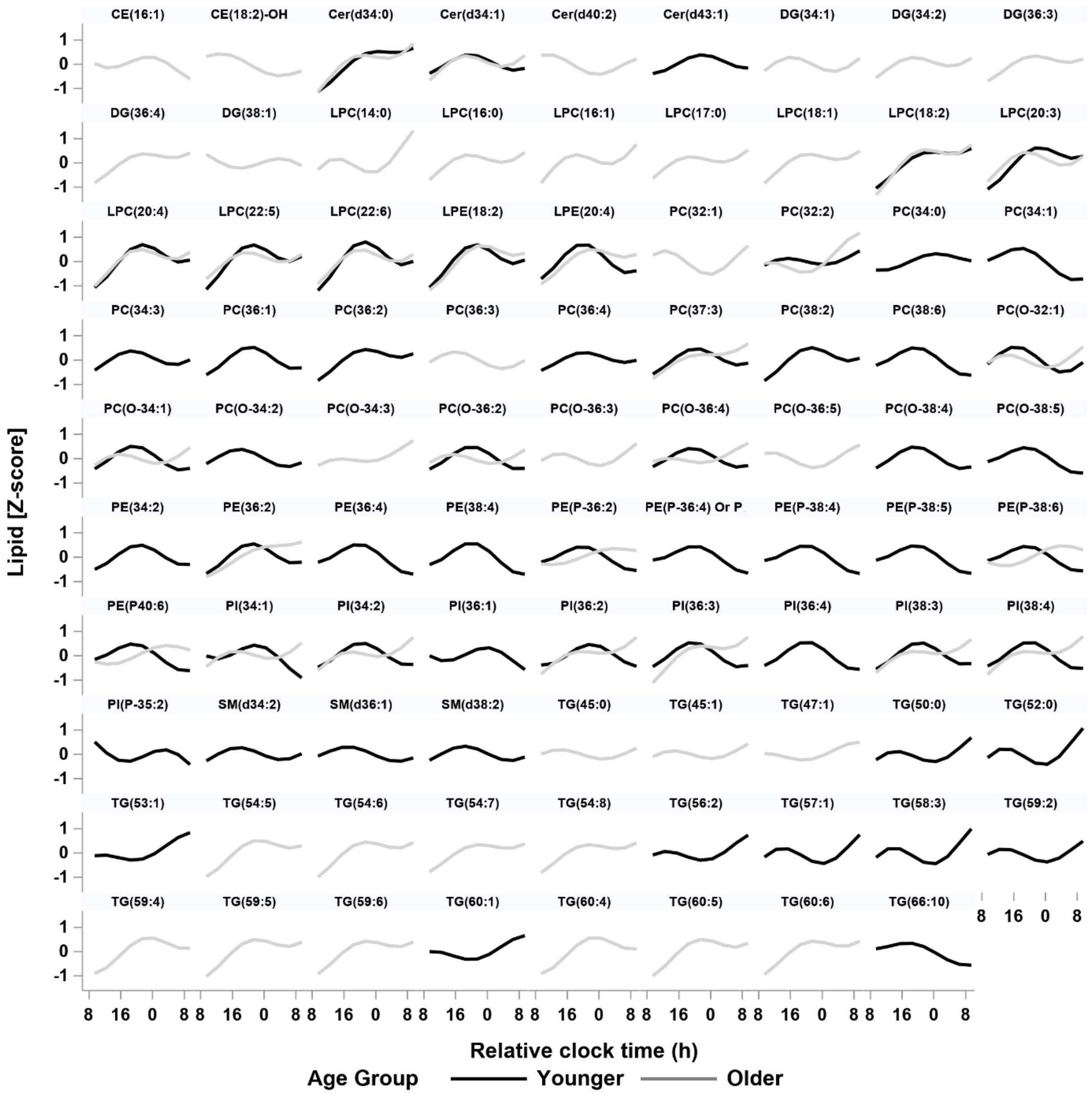

Supplementary Figure 3. Predicted time-course profiles calculated from group-mean data across the 27-h constant routine are shown for each circadian lipid species for the younger (**—**, in black) and older (—, in gray) age groups. Data are plotted to a relative clock time with wake time assigned a value of 8:00 am.

Supplementary Figure 4

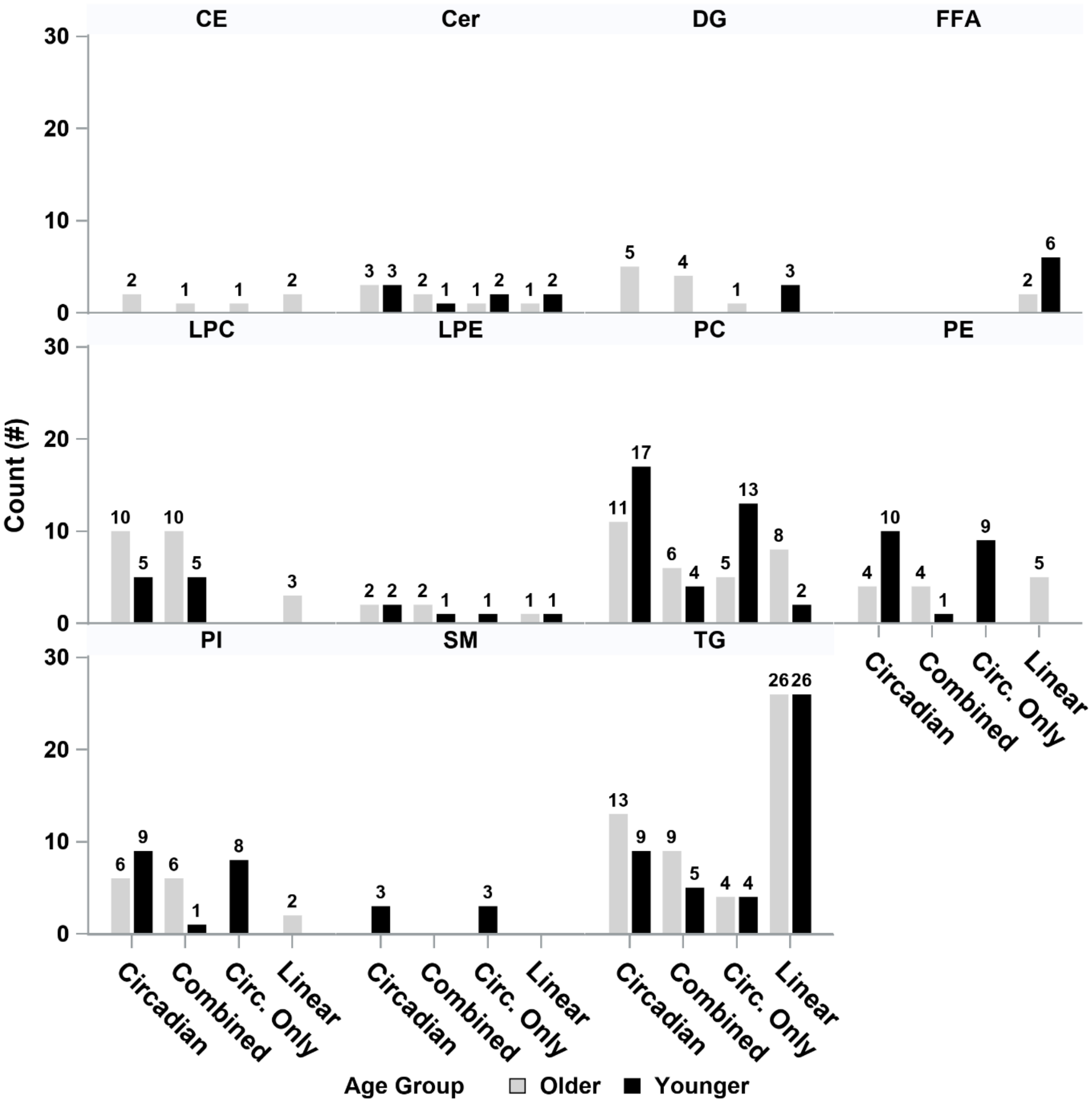

Supplementary Figure 4. Prevalence of circadian and linear lipid profiles during the 27-h constant routine in the younger- and older-age groups. Circadian profiles were further dichotomized to be “Combined” when both the sinusoidal and linear terms were significant or only circadian (Circ. only) when the sinusoidal but not the linear term was significant in the Cosinor regression.

Supplementary Figure 5

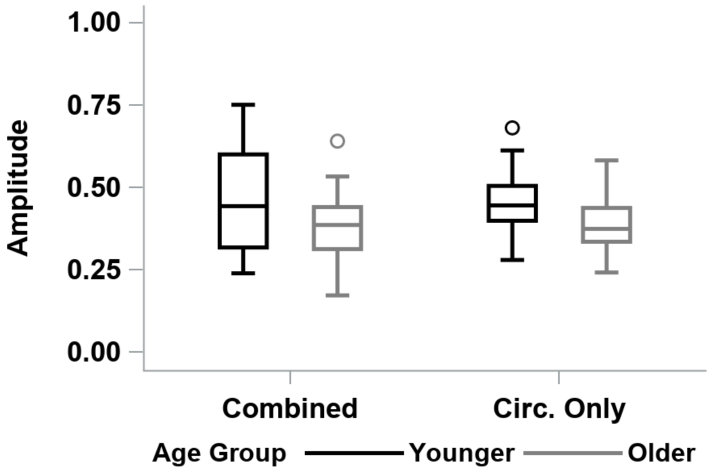

Supplementary Figure 5. The box and whisker plots show the median, 25th and 75th percentile (box limits), the 10th and 90th percentiles (whiskers), and outlier points (○, unfilled circle) for amplitude estimates for circadian lipids for the younger and older age groups derived from group-mean data. Circadian profiles were dichotomized to be “Combined” when both the sinusoidal and linear terms were significant or only circadian (Circ. only) when the sinusoidal but not the linear term was significant in the Cosinor regression.

Supplementary Figure 6

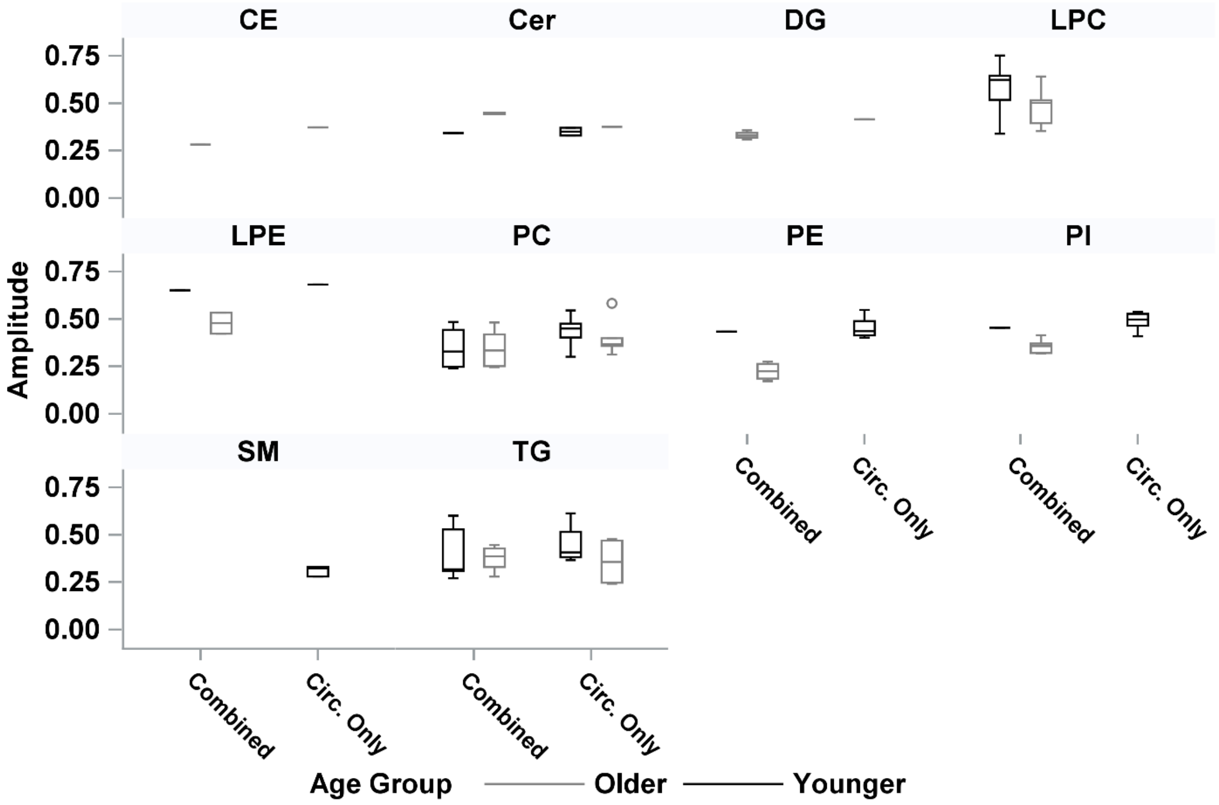

Supplementary Figure 6. The box and whisker plots show the median, 25th and 75th percentile (box limits), the 10th and 90th percentiles (whiskers), and outlier points (○, unfilled circle) for amplitude estimates for circadian lipids in each lipid subclass for the younger and older age groups derived from group-mean data. Circadian profiles were dichotomized to be "Combined" when both the sinusoidal and linear terms were significant or only circadian (Circ. only) when the sinusoidal but not the linear term was significant in the Cosinor regression.

Supplementary Figure 7

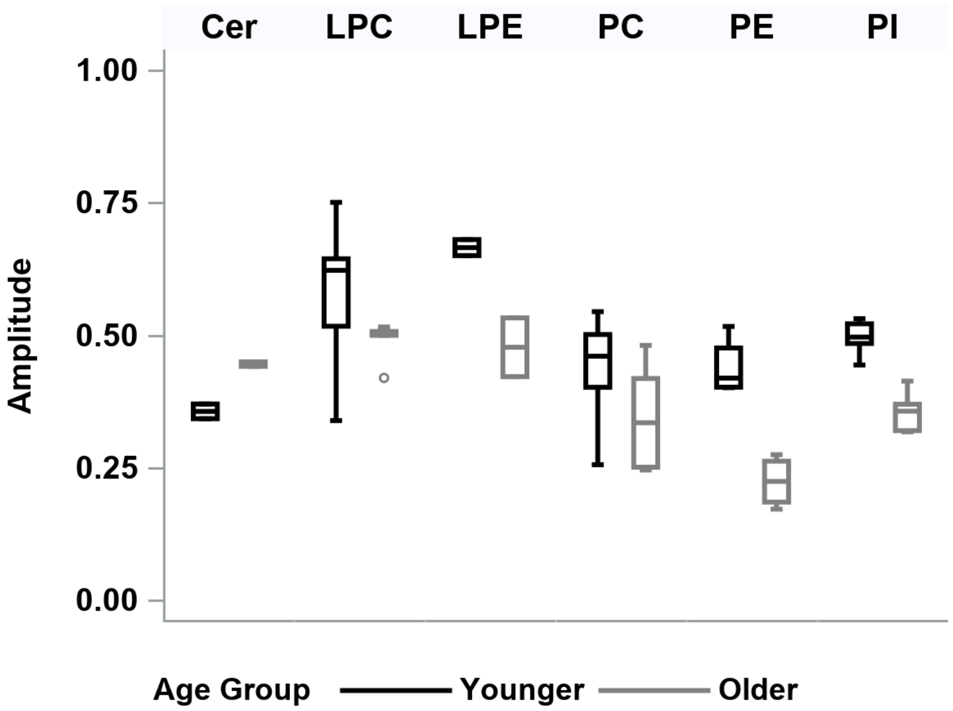

Supplementary Figure 7. The box and whisker plots show the median, 25th and 75th percentile (box limits), the 10th and 90th percentiles (whiskers), and outlier points (○, unfilled circle) for amplitude estimates for circadian lipids in each lipid subclass for the younger and older age groups derived from individual-level data. Circadian profiles were dichotomized to be “Combined” when both the sinusoidal and linear terms were significant or only circadian (Circ. only) when the sinusoidal but not the linear term was significant in the Cosinor regression.
