# Supplementary material for: Age-related changes in circadian regulation of the human plasma lipidome
Source: Commun Biol. 2023 Jul 20;6:756. doi: 10.1038/s42003-023-05102-8 (PMC10359364; doi:10.1038/s42003-023-05102-8)
Supplement: Supplementary file 5 — Reporting Summary [file 42003_2023_5102_MOESM5_ESM.pdf]

nature portfolio

Corresponding author(s): Bruce Kristal
Last updated by author(s): Sep 22, 2022

# Reporting Summary

Nature Portfolio wishes to improve the reproducibility of the work that we publish. This form provides structure for consistency and transparency in reporting. For further information on Nature Portfolio policies, see our Editorial Policies and the Editorial Policy Checklist.

## Statistics

For all statistical analyses, confirm that the following items are present in the figure legend, table legend, main text, or Methods section.

| n/a | Confirmed | |
|---|---|---|
| ☐ | ☒ | The exact sample size (*n*) for each experimental group/condition, given as a discrete number and unit of measurement |
| ☐ | ☒ | A statement on whether measurements were taken from distinct samples or whether the same sample was measured repeatedly |
| ☐ | ☒ | The statistical test(s) used AND whether they are one- or two-sided<br>*Only common tests should be described solely by name; describe more complex techniques in the Methods section.* |
| ☐ | ☒ | A description of all covariates tested |
| ☐ | ☒ | A description of any assumptions or corrections, such as tests of normality and adjustment for multiple comparisons |
| ☐ | ☒ | A full description of the statistical parameters including central tendency (e.g. means) or other basic estimates (e.g. regression coefficient) AND variation (e.g. standard deviation) or associated estimates of uncertainty (e.g. confidence intervals) |
| ☐ | ☒ | For null hypothesis testing, the test statistic (e.g. *F*, *t*, *r*) with confidence intervals, effect sizes, degrees of freedom and *P* value noted<br>*Give P values as exact values whenever suitable.* |
| ☒ | ☐ | For Bayesian analysis, information on the choice of priors and Markov chain Monte Carlo settings |
| ☐ | ☒ | For hierarchical and complex designs, identification of the appropriate level for tests and full reporting of outcomes |
| ☒ | ☐ | Estimates of effect sizes (e.g. Cohen's *d*, Pearson's *r*), indicating how they were calculated |

*Our web collection on statistics for biologists contains articles on many of the points above.*

## Software and code

Policy information about availability of computer code

| | |
|---|---|
| Data collection | Feature selection (framing) and relative quantitation (based on peak area) was done using SIEVE v 2.1 (Thermo Fisher Scientific and Vast Scientific, Cambridge, MA). |
| Data analysis | All analyses were performed using SAS 9.4 (SAS Institute Inc., Cary, NC, USA). |

For manuscripts utilizing custom algorithms or software that are central to the research but not yet described in published literature, software must be made available to editors and reviewers. We strongly encourage code deposition in a community repository (e.g. GitHub). See the Nature Portfolio guidelines for submitting code & software for further information.

## Data

Policy information about availability of data

All manuscripts must include a data availability statement. This statement should provide the following information, where applicable:

- Accession codes, unique identifiers, or web links for publicly available datasets
- A description of any restrictions on data availability
- For clinical datasets or third party data, please ensure that the statement adheres to our policy

The data that support the findings of this study are available on request from the corresponding author, (BSK) or lead author (SAR). The data are not publicly available due to containing information that could compromise the privacy of research participants.

## Human research participants

Policy information about studies involving human research participants and Sex and Gender in Research.

| | |
|---|---|
| Reporting on sex and gender | Self-reported data on sex was collected from all participants. The study included nine females and 15 males. All participants were consented to collect individual-level data. Sex of each participant is reported in Supplemental Table 1. |
| Population characteristics | The study included 12 healthy young (4 females, mean±SD age: 23.5±3.9 years) and 12 healthy middle-aged (5 females, 58.3 ±4.2 years) individuals. All participants were determined to be healthy by physical, psychological, and ophthalmologic clinical exams. |
| Recruitment | Participants were recruited from the local population (i.e., Boston and surrounding areas) by means of print and online advertisements. |
| Ethics oversight | The study was reviewed and approved by the Partners Human Research Committee (Boston, Massachusetts, USA), and participants provided written informed consent prior to study. |

Note that full information on the approval of the study protocol must also be provided in the manuscript.

# Field-specific reporting

Please select the one below that is the best fit for your research. If you are not sure, read the appropriate sections before making your selection.

☒ Life sciences ☐ Behavioural & social sciences ☐ Ecological, evolutionary & environmental sciences

For a reference copy of the document with all sections, see nature.com/documents/nr-reporting-summary-flat.pdf

# Life sciences study design

All studies must disclose on these points even when the disclosure is negative.

| | |
|---|---|
| Sample size | Sample size was not determined using statistical analysis. We used data from two studies conducted in the same facility with identical procedures relevant to this work (i.e., constant routine protocols). The study by Scheuermaier et al. (J Biol Rhythms 2019) included 12 healthy older participants, data from all of whom were included in the study. The study by Rahman et al., (JCI Insight 2017) included 26 healthy younger participants, and data from 12 healthy participants were selected at random for inclusion in the current study. |
| Data exclusions | All available data were included in the analyses. |
| Replication | Results related to differences in acrophase and amplitude of circadian rhythms in lipids between younger and older groups have not been replicated. The presence of circadian rhythms in plasma lipids and their acrophase times are consistent with prior reports (e.g., Chua et al., PNAS 2013, Dallmann et al., PNAS 2012). The results related to differences in circadian phase and amplitude of centrally controlled rhythms with aging are consistent with previous reports (e.g., Czeisler et al., 1992 Lancet, Zeitzer et al., Am J Med 1999). |
| Randomization | All participants completed the same study intervention (i.e., constant routine protocol). Data for older and younger cohorts were collected at different times. Data for the younger cohort was from 12 healthy participants randomly selected from 26 young healthy participants who completed the primary study. |
| Blinding | All participants completed the same study intervention (i.e., constant routine protocol) and were not blinded from the study conditions. Per study design and IRB approval, participants were not made aware of the actual duration of the constant routine protocol until the end of the data collection and were studied in an environment with no obvious time cues. Investigators could not be blinded from knowing the age of participants (principal determinant of study grouping) as it was an inclusion criteria for the original studies for each age cohort. |

# Reporting for specific materials, systems and methods

We require information from authors about some types of materials, experimental systems and methods used in many studies. Here, indicate whether each material, system or method listed is relevant to your study. If you are not sure if a list item applies to your research, read the appropriate section before selecting a response.

## Materials & experimental systems

| n/a | Involved in the study | |
|---|---|---|
| ☐ | ☒ | Antibodies |
| ☒ | ☐ | Eukaryotic cell lines |
| ☒ | ☐ | Palaeontology and archaeology |
| ☒ | ☐ | Animals and other organisms |
| ☒ | ☐ | Clinical data |
| ☒ | ☐ | Dual use research of concern |

## Methods

| n/a | Involved in the study | |
|---|---|---|
| ☒ | ☐ | ChIP-seq |
| ☒ | ☐ | Flow cytometry |
| ☒ | ☐ | MRI-based neuroimaging |

# Antibodies

Antibodies used

Melatonin concentration in the younger individuals was determined by double-antibody radioimmunoassay with the Kennaway G280 antiserum (Specialty Assay Research Core Laboratory, Brigham and Women's Hospital). Melatonin concentration in the older individuals was determined using the Bühlmann Melatonin Direct RIA kit (Bühlmann Laboratories, Schönenbuch, Switzerland) and the assays conducted by Solidphase, Inc., (Portland, ME).

Validation

Validation was performed by each independent laboratory.
